# Genome-wide analysis of p53 transcriptional programs in B cells upon exposure to genotoxic stress *in vivo*

**DOI:** 10.18632/oncotarget.5232

**Published:** 2015-09-05

**Authors:** Claudia Tonelli, Marco J. Morelli, Salvatore Bianchi, Luca Rotta, Thelma Capra, Arianna Sabò, Stefano Campaner, Bruno Amati

**Affiliations:** ^1^ Department of Experimental Oncology, European Institute of Oncology (IEO), Milan, Italy; ^2^ Center for Genomic Science of IIT@SEMM, Fondazione Istituto Italiano di Tecnologia (IIT), Milan, Italy

**Keywords:** p53, transcription, DNA damage, B cells, chromatin

## Abstract

The tumor suppressor p53 is a transcription factor that coordinates the cellular response to DNA damage. Here we provide an integrated analysis of p53 genomic occupancy and p53-dependent gene regulation in the splenic B and non-B cell compartments of mice exposed to whole-body ionizing radiation, providing insight into general principles of p53 activity *in vivo*. In unstressed conditions, p53 bound few genomic targets; induction of p53 by ionizing radiation increased the number of p53 bound sites, leading to highly overlapping profiles in the different cell types. Comparison of these profiles with chromatin features in unstressed B cells revealed that, upon activation, p53 localized at active promoters, distal enhancers, and a smaller set of unmarked distal regions. At promoters, recognition of the canonical p53 motif as well as binding strength were associated with p53-dependent transcriptional activation, but not repression, indicating that the latter was most likely indirect. p53-activated targets constituted the core of a cell type-independent response, superimposed onto a cell type-specific program. Core response genes included most of the known p53-regulated genes, as well as many new ones. Our data represent a unique characterization of the p53-regulated response to ionizing radiation *in vivo*.

## INTRODUCTION

The tumor suppressor p53 is central to the regulation of cell fate in response to a wide variety of cellular insults including DNA damage, oncogene overexpression, hypoxia and oxidative stress, among others. Once activated, p53 can promote responses including apoptosis, senescence, cell cycle arrest and metabolic reprogramming [1, 2]. Consistent with its critical role in the regulation of these tumor suppressive programs, the *TP53* gene is frequently mutated in human cancers. Most of these mutations occur in the exons encoding the DNA binding domain of the protein, highlighting how crucial p53's function as a transcription factor is for its activity [3].

In spite of extensive research, our understanding of the detailed molecular mechanisms activated by p53 remains incomplete. Previous genome-wide studies of p53 chromatin occupancy using chromatin immunoprecipitation (ChIP) followed by hybridization (ChIP-chip) [4–7], paired-end ditag PET sequencing (ChIP-PET) [8], deep sequencing (ChIP-Seq) [9–28] or exonuclease treatment (ChIP-exo) [29] coupled with gene expression data helped in filling in part this gap. First of all, the p53 binding motif, or p53 response element (p53-RE), was better characterized and software tools for its identification were developed [4, 8, 16]: the p53-RE is defined as two decameric half sites, each with the consensus 5′-RRRCWWGYYY-3′ (R = purine, W = A or T, Y = pyrimidine), prevalently placed one next to the other or, with lower incidence, separated by a spacer of 1-13 bp [30–32]. Second, novel pathways and regulatory connections were discovered. For example, p53 regulates differentiation of mouse embryonic stem cells through the induction of genes in the Wnt signaling pathway [5] and induces an autophagy program in mouse embryonic fibroblasts in response to the DNA damaging agent doxorubicin [14]. Finally, it was observed that p53 plays a role at large distances from transcription start sites (TSSs). Distal p53 binding sites can reside either in active enhancers [28, 33] or in closed chromatin [24, 25], and have been associated with the regulation of non-coding RNA species, such as microRNAs [34] and long intergenic non-coding RNAs [23, 26]. Here, to characterize the p53-dependent programs *in vivo*, we compared the transcriptional responses to whole-body ionizing radiation (IR) in B cells and a T cell-enriched population from either wild-type or *Trp53^−/−^* mice. We chose to use DNA damage as a p53 activating stimulus because *Trp53* null thymocytes were previously shown to be deficient in radiation-induced apoptosis, demonstrating the importance of p53 in the response to genotoxic stress [35, 36]. Our study revealed novel components of the p53-regulated network. Moreover, we showed that p53 binding to the canonical response element as p53 binding strength associates with p53-dependent gene induction, but not repression. To our knowledge, this dataset represents the first whole genome mapping of p53 binding and gene expression profiles in response to stress performed *in vivo*.

## RESULTS

**Figure 1:**
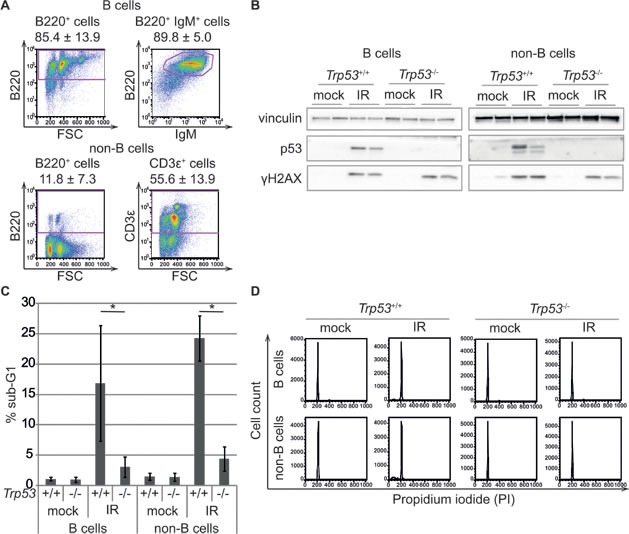
Phenotypic characterization of the experimental model **A.** FACS analysis of splenic cells after sorting for the B220 isoform of CD45. B cells (B220^+^ cells) are analyzed for B220 and IgM expression and non-B cells (B220^−^ cells) for B220 and CD3ε expression. The data represent the average ± standard deviation of flow cytometric analyses of twelve mice. **B.** p53 stabilization is induced by DNA damage in B and non-B cells from *Trp53^+/+^* mice treated with 7 Gy of irradiation (IR) compared to control animals (mock) and *Trp53^−/−^* animals. Four hours after IR, spleens are harvested from each mouse and B and non-B cells are purified by cell sorting for B220. Protein lysates from individual mouse cells are subjected to western blot analysis with antibodies specific for the indicated proteins. Phosphorylation of H2AX is shown as a positive control for DNA damage. **C.** The accumulation of cells with sub-G1 DNA content is examined using flow cytometry in B and non-B cells from control (mock) and irradiated (IR) *Trp53^+/+^* and *Trp53^−/−^* mice. The reported values represent the average ± standard deviation of three biological replicates. *p < 0.05 (Student's *t*-test) **D.** Representative flow cytometric analysis of DNA content (Propidium iodide staining) of B and non-B cells from control (mock) and irradiated (IR) *Trp53^+/+^* and *Trp53^−/−^* mice.

**Figure 2:**
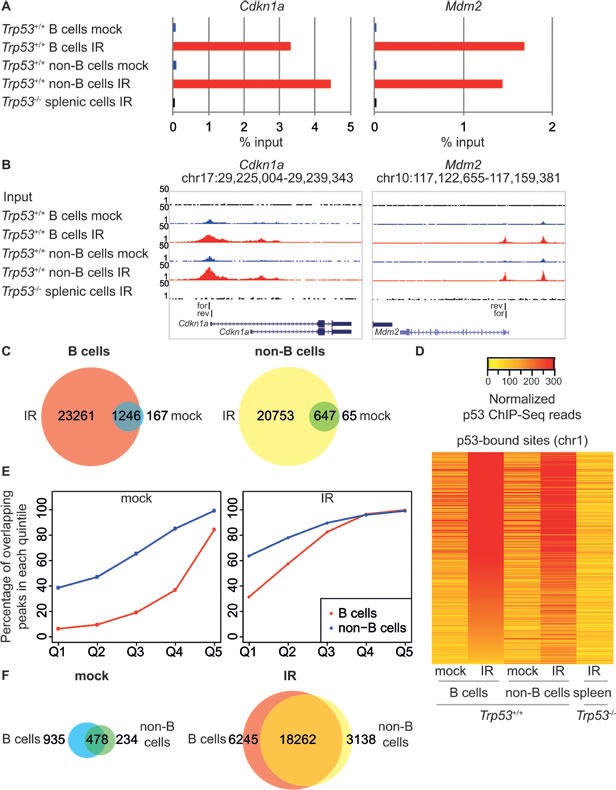
Genome-wide analysis of p53 binding in response to IR **A.** p53 ChIP-qPCR analysis of the *Cdkn1a* and *Mdm2* promoters. ChIP data are quantified as percentage of input. **B.** UCSC Genome Browser views of p53 ChIP-Seq peaks in B and non-B cells from control (mock; blue tracks) and irradiated (IR; red tracks) *Trp53^+/+^* animals, negative control ChIP-Seq in splenic cells from irradiated *Trp53^−/−^* animals and Input control data (black tracks) at the same loci as in A. The same scale is used for all examples presented (1-50 ChIP-Seq tags). The region amplified in ChIP-qPCR is indicated (for: forward primer; rev: reverse primer). **C.** Overlap between p53 binding sites in control (mock) and irradiated (IR) B and non-B cells from *Trp53^+/+^* mice. **D.** Heatmap showing library size-normalized ChIP-seq read counts for all p53-bound sites at chromosome 1 in B and non-B cells from control (mock) and irradiated (IR) *Trp53^+/+^* mice and total splenic cells from irradiated *Trp53^−/−^* mice, as indicated. Peaks are ranked from top to bottom by reads density in irradiated B cells. **E.** Percentage of overlapping p53 peaks of different enrichment levels between mock (left panel) and irradiated (right panel) B and non-B cells. Peaks are grouped based on the enrichment values into quintiles (Q1-Q5); for each quintile the percentage of overlapping peaks is indicated. **F.** Overlap between p53 binding sites in B and non-B cells from control (mock) and irradiated (IR) *Trp53^+/+^* mice.

We compared p53 binding and mRNA expression profiles in response to whole-body ionizing radiation in mouse splenic mature B cells and in a T cell-enriched population constituted by the rest of the cells in the organ (hereafter “non-B cells”, Figure 1A), from either wild-type or *Trp53^−/−^* mice. DNA damage induced p53 stabilization and p53-dependent apoptosis (Figure 1B, 1C), without affecting the distribution of the cells in the different cell cycle phases, as these cells were mainly quiescent and remained in G_0_/G_1_ following IR (Figure 1D). Targeted ChIP analysis and ChIP-seq profiling confirmed IR-induced binding of p53 to the known target loci *Cdkn1a* and *Mdm2*, with no signal detected in total splenic cells from irradiated *Trp53^−/−^* mice, thus demonstrating the specificity of the p53 antibody (Figure 2A, 2B). Irradiation greatly increased the total number of p53 binding sites in either mature B cells and non-B cells (from around 1,000 to more than 20,000). Virtually all of the sites identified in the non-irradiated samples were also retrieved in the irradiated ones, where they constituted some of the most enriched peaks (Figure 2C, 2D). The overlap in the p53 peaks between B and non-B cells increased with peak enrichment (Figure 2E), reaching 75-85% overall in the irradiated samples (Figure 2F), indicative of very similar p53 binding profiles in the two different cell populations.

**Figure 3:**
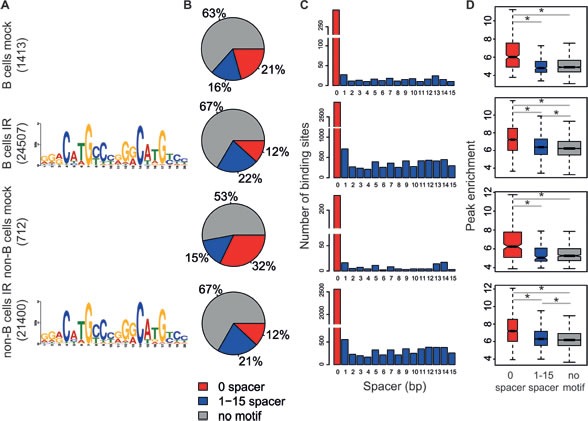
Motif analysis of p53 binding sites The analysis presented here was performed as described in [15]. **A.** Consensus p53-response elements identified by MEME *de novo* motif analysis on the top 1000 p53-binding sites for each sample. **B.** Frequency of p53-binding sites containing no motif (grey), motif with zero spacer between the two decameric half sites (red), motif with a 1-15 nt spacer (blue). **C.** Distribution of spacer lengths between p53-RE half sites. **D.** Boxplots of the peak enrichment of p53-binding sites as a function of the associated motif (color code as in B). The boxes were drawn with widths proportional to the square-roots of the number of observations in the groups. *p < 10^−7^ (Student's *t*-test).

A *de novo* motif analysis using MEME [37] on the 1000 most highly enriched p53 peaks identified the p53 consensus in the irradiated samples (Figure 3A), closely resembling the one observed in previous genome-wide studies [4, 8, 13, 15]. Using the p53 matrix derived by MEME, we scanned all p53 ChIP-Seq peaks with FIMO [38] and checked for the occurrence of the inferred p53 motif accounting also for a spacer of 1-15 nucleotides (nt) between the two decameric half sites. The unsplit p53-RE was identified in approximately 12 to 32% of the binding sites and another 15 to 22% presented the motif with a 1-15 nt spacer (Figure 3B, 3C). About one quarter of these sites included multiple copies of the p53-RE, as observed in [14, 29]. In the irradiated samples, p53-REs with no spacer constituted the highest affinity sites followed by motifs with a spacer, as indicated by peak intensity (Figure 3D) [15]. The remaining sites without an identifiable motif showed the lowest binding levels: we surmise that p53 may associate with those sites through either non-sequence specific DNA binding, protein-protein interactions, or chromatin looping.

**Figure 4:**
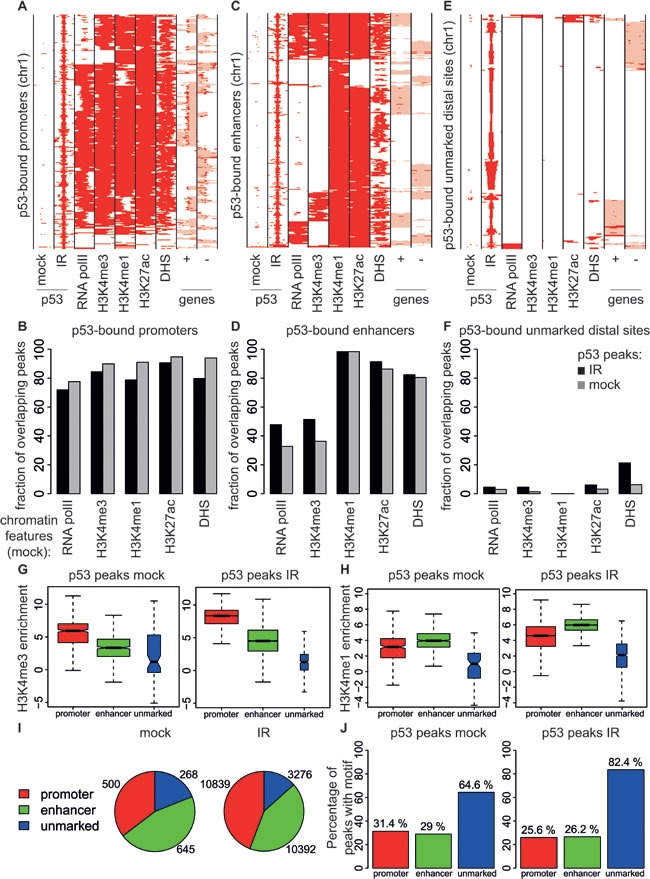
Genomic distribution of p53 binding sites in B cells **A.**, **C.**, **E.**. Heatmaps showing the distribution of p53 at **A**. annotated promoters (−5/+2 kb from the TSS) **C.**, enhancers (H3K4me1-positive regions, not overlapping with promoters) and **E.** unmarked distal regions (everything else). Each row represents a different genomic interval (2 kb width centered on p53 peaks). The panels include every region in chromosome 1 that was identified as p53-bound by ChIP-seq in the B cells. For the same intervals, the distributions of RNA polII, H3K4me3, H3K4me1, H3K27ac, DHS in non-irradiated B cells and annotated genes (exons in red, introns in pink; + sense, -antisense strand) are also shown. **B.**, **D.**, **F.**. As in [24] these panels show the percentage of p53 peaks overlapping with RNA polII, H3K4me3, H3K4me1, H3K27ac, DHS peaks at **B.** promoters **D.** enhancers and **F.** unmarked distal regions. **G.**, **H.** Box plots showing the enrichment of H3K4me3 or H3K4me1 peaks overlapping with p53 binding sites at promoters (red boxes), enhancers (green boxes), and unmarked distal regions (blue boxes) in B cells from control (mock) and irradiated (IR) wild-type mice. The boxes were drawn with widths proportional to the square-roots of the number of observations in the groups. **I.** Number of p53 binding sites at promoters (red), enhancers (green), and unmarked distal regions (blue) in B cells from control (mock) and irradiated (IR) wild-type mice. **J.** Percentage of p53 peaks at promoters (red bars), enhancers (green bars) and unmarked distal regions (blue bars) containing the p53 motif.

To better characterize the p53 binding sites, we classified them according to their position in the genome and chromatin-associated features, using RNA polymerase II (RNA polII) and the active histone marks H3K4me3, H3K4me1 and H3K27ac [39] that we had previously mapped in the same population of B cells in non-irradiated mice [40], as well as DNaseI hypersensitive sites (DHS). Virtually all promoter-proximal p53 binding sites shared these features, indicating that p53 associated with promoters that pre-existed in an active and accessible state in non-irradiated B cells (Figure 4A, 4B, 4G, 4H). 75% of the p53-bound distal sites in irradiated B cells showed high H3K4me1 and low H3K4me3 levels and also presented H3K27ac, characteristic of active enhancers [39] (Figure 4C, 4D, 4G, 4H). The remaining distal sites lacked any of the investigated chromatin marks and were located in regions protected from DNase I digestion, indicative of closed chromatin prior to irradiation (Figure 4E, 4F, 4G, 4H). In both control and irradiated B cells, 15 to 20% of the p53 peaks were unmarked distal sites and the remaining ones were similarly distributed between promoters and enhancers (Figure 4I). Remarkably, these unmarked distal sites were the most highly enriched for the p53 consensus (Figure 4J). Together with their compact state and the lack of active chromatin marks prior to p53 activation, these sites are reminiscent of “proto-enhancers”, at which p53 was proposed to act as a pioneer transcription factor: this will require further investigation, however, since no opening of the chromatin was observed upon p53 binding at those sites [24].

**Figure 5:**
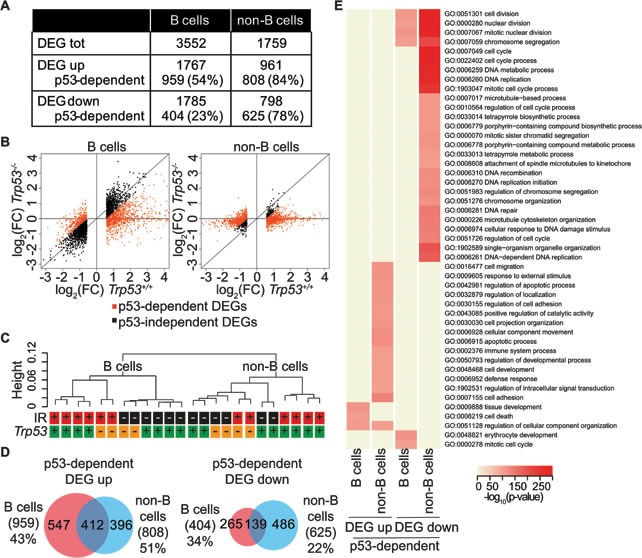
IR-regulated genes **A.** Summary of IR-regulated genes in B and non-B cells from *Trp53^+/+^* mice (DEG UP: q-value < 0.05, log_2_ of fold change > 0.58; DEG DOWN: q-value < 0.05, log_2_ of fold change < −0.58). The number and the percentage of p53-dependent DEGs are reported. **B.** Scatter plot of the log_2_ of fold change values of all DEGs as estimated by RNA-Seq analysis in irradiated B and non-B cells relative to controls in *Trp53^+/+^* mice (x-axis) and in *Trp53^−/−^* mice (y-axis). Black dots indicate p53-independent DEGs, orange dots indicate p53-dependent DEGs. p53-dependent genes are defined as follows: all up-regulated genes in *Trp53*^+/+^ cells with a fold induction upon IR at least 1.5 times higher than in *Trp53^−/−^* cells and all down-regulated genes in *Trp53*^+/+^ cells with a fold repression upon IR at least 1.5 times lower than in *Trp53^−/−^* cells. **C.** Unsupervised hierarchical clustering (computed with Spearman rank correlation coefficients) of RNA-Seq RPKM of all expressed genes (RPKM > 1 in at least one sample) in B and non-B cells from control (black) or irradiated (red) *Trp53^+/+^* (green) and *Trp53^−/−^* (orange) animals. **D.** Overlap between p53-dependent DEGs in B and non-B cells. The percentages indicate the fraction of common DEGs for each cell type. **E.** Gene Ontology terms enriched in up- and down-regulated p53-dependent genes in B and non-B cells following IR exposure relative to controls. GO terms significantly enriched (Fisher's test *p*-value < 1*10^−10^) in at least one gene set were selected (rows in the heatmap) and the *p*-values for each GO term in each gene set (columns) were color-coded as indicated. Only GO terms in the biological process ontology that are assigned to less than 2,000 and more than 10 genes in the mouse genome are considered.

We used RNA-Seq to profile gene expression before and after IR exposure. Normalizing the mean expression values of irradiated samples relative to those of unstressed controls yielded 3552 and 1759 differentially expressed genes (DEGs) in B and non-B cells, respectively, with roughly equal proportions of up- and down-regulated genes in either cell type (Figure 5A). Based upon loss of the response in *Trp53* knockout animals, 38% and 81% of the DEGs in B and non-B cells, respectively, were classified as p53-dependent (Figure 5B). Unsupervised hierarchical clustering of the RNA-Seq data showed a clear separation of B and non-B cells samples, indicating that cell type was the major determinant of the expression profiles (Figure 5C) [41]. IR and genotype also contributed in shaping the transcriptome, as most evident for B cells, in which irradiated and control samples formed distinct clusters, further sub-divided between *Trp53*^+/+^ and *Trp53^−/−^* samples. In non-B cells, in which the response to stress was primarily p53-dependent, the *Trp53^−/−^* irradiated samples clustered with non-irradiated control samples. These features may largely be due to the more heterogeneous nature of the non-B cell population. In contrast to the p53 binding profiles, which were remarkably similar between B and non-B cells (Figure 2F), the p53-dependent programs activated by IR exposure were more divergent, implying the involvement of other factors, such as cofactors or post-translational modifications of p53, in determining the transcriptional response in a cell type-specific manner (Figure 5D). A biological process Gene Ontology (GO) analysis indicated that the main pathways that were up-regulated in a p53-dependent manner both in B and non-B cell were related to cell death, while terms related to cell division and cell cycle were enriched in the repressed genes, consistent with the known biological activities of p53 [42].

**Figure 6:**
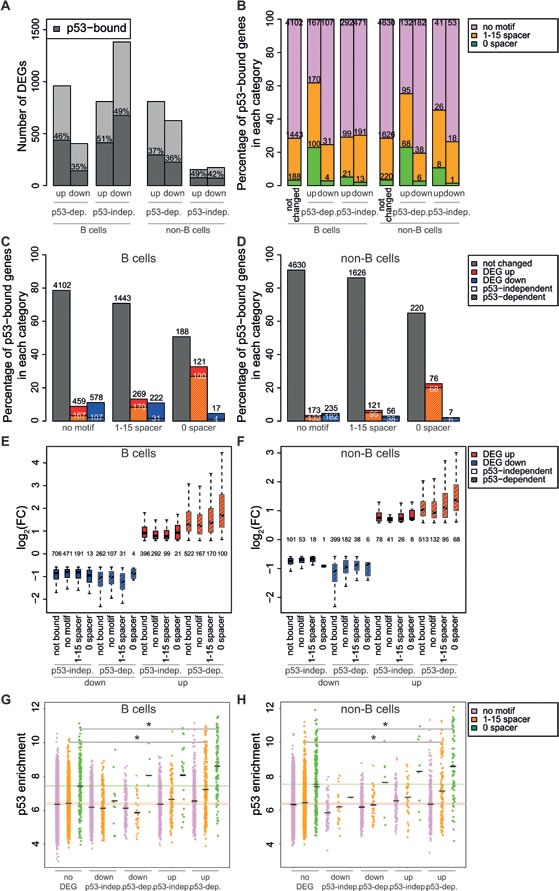
p53 binding intensity and the presence of the unsplit p53 motif associate with gene induction **A.** Bar plot showing the numbers of differentially expressed genes (DEGs) as a function of their regulatory mode in B or non-B cells, as indicated (up, down, p53-dependent or independent). The % of p53-bound genes (dark grey) is indicated within each bar. **B.** Percentage p53-bound genes containing no motif, the motif with a 1-15 nt spacer or no spacer, as a function of their regulatory mode, as indicated. The absolute number of genes in each category is shown within the bars. **C.**, **D.** Percentage p53-bound genes in each regulatory category, as a function of the associated motif, as indicated. Plain bars: p53-independent; dashed: p53-dependent. **E.**, **F.** Box plot showing the fold-change (log2 FC) of IR-regulated genes as a function of their regulatory category and p53-binding motif, as indicated. The number of genes in each category is reported. **G.**, **H.** Dot plot showing the enrichment of p53 peaks in promoter regions following irradiation, as a function of their regulatory category and p53-binding motif, as indicated. The black lines represent the mean. * *p* < 10^−5^ (Student's t-test).

To determine which of the IR-regulated genes were direct p53 targets, we compared p53 binding and expression profiles (Figure 6A). 35% to 51% of the up- and down-regulated genes in either B and non-B cells had a p53 binding site in the promoter region (−5 to +2kb from the TSS). p53 peaks were detected, with similar frequency, also at the promoter of p53-independent DEGs, implying that mere p53 binding was not predictive of transcriptional regulation. However, transcriptional regulation did correlate with sequence-specific DNA binding, as indicated by the differential frequencies of the p53-RE: while the motif with a spacer sequence was present at the promoter of both p53-dependent and -independent DEGs, the unsplit motif was enriched primarily nearby the TSS of p53-dependent up-, but not down-regulated genes (Figure 6B). Thus, our data indicate that activation, but not repression, is mediated through p53 binding to the unsplit p53-RE. In a complementary manner, p53 binding to the unsplit motif was more predictive of p53-dependent activation (Figure 6C, 6D). These results suggest that p53 directly regulates the expression of the genes that it binds via the unsplit p53-RE. Of note, p53-dependent genes were the most significantly regulated (either up or down) and among the up-regulated genes those with the unsplit p53 motif showed increased induction by IR compared to the other categories (Figure 6E, 6F). Finally, among the p53 peaks with the motif, those at p53-activated promoters showed on average the highest binding intensities, although with a significant level of overlap with non-regulated promoters (Figure 6G, 6H), showing that binding strength *per se* is not predictive of regulation. We speculate that the genes bound by p53 at the p53-RE that were not identified as differentially expressed in response to DNA damage may either (i.) be activated in a minor fraction of cells, resulting in non-significant regulation at the population level, or (ii.) depend upon additional signals or factors absent in the condition studied here.

**Figure 7:**
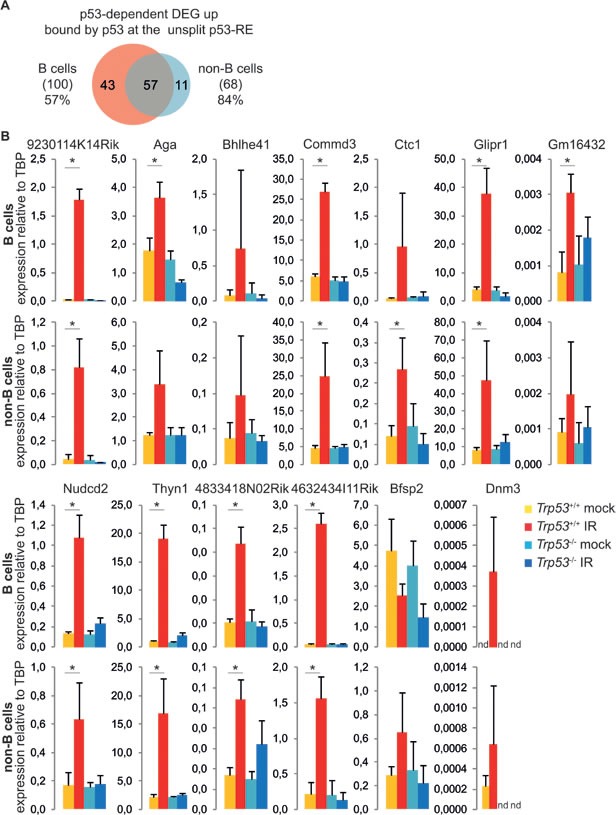
Validation of new p53 targets **A.** Overlap between p53-dependent up-regulated genes having a p53 binding site containing the unsplit motif in B and non-B cells. The percentages indicate the fraction of common DEGs for each cell type. **B.** Real-time RT-PCR analysis of B and non-B cells from control (mock) and irradiated (IR) *Trp53*^+/+^ and *Trp53^−/−^* mice shows p53-dependent up-regulation of the indicated genes. Slc22a12 could not be detected by Real-time RT-PCR. The reported values represent the average ± standard deviation of three biological replicates. *p < 0.05 (Student's *t*-test).

Finally, the p53-dependent IR-induced genes with a bound, unsplit p53-RE showed a strong overlap among B and non-B cells, suggesting that these genes constitute the core of a cell type-independent p53 response (Figure 7A). We compared our list of p53-dependent p53-bound DEGs in B and non-B cells with previously described p53 targets (Supplementary Table 1) [8, 12–16, 32, 43, 44]: 14 genes of the cell type-independent p53 response represented new p53-responsive genes, most of which were validated by Real-time RT-PCR (Figure 7B). Further experimental validation will be necessary to understand the functional role of these genes in the response to IR.

## DISCUSSION

To identify the genes regulated by p53 *in vivo*, we exposed mice to whole-body ionizing radiation and determined whole-genome p53 binding and gene expression profiles in splenic B and non-B cell populations. Our data revealed that p53 was already bound to a small fraction of its genomic targets in the absence of any extrinsic stress, consistent with recent observations in the developing kidney [17]. The binding of p53 to chromatin under basal conditions was previously reported in normal (non-tumorigenic) cells grown *in vitro* [5, 12, 14], but culture-associated stress may have constituted an activating stimulus in those conditions. *In vivo* data in the whole organism thus confirm the association of p53 with chromatin in the absence of external stress stimuli: this may reflect a poising mechanism allowing fast responses to stress, as previously shown for *Cdkn1a* [45, 46], or the presence of cell-intrinsic stresses, such as replicative or oxidative stress. This notwithstanding, irradiation greatly increased the total number of binding sites in either B or non-B cells, consistent with an acute activation of p53 in those conditions.

p53 binding sites were distributed between promoters and distant loci, most of the distal peaks showing the characteristic features of enhancers (high H3K4me1 and low H3K4me3) [39]. Enhancers have been shown to play a central role in p53-mediated gene regulation: on the one hand, p53 was described to interfere with enhancer activity to repress the expression of target genes in mouse embryonic stem cells [12]; on the other, it was shown to induce the expression of enhancer RNAs to activate target genes in fibroblasts [33]. In support of an activating rather than a repressing role at enhancers, p53 drove enhancer RNA transcription in the vicinity (<25 kb) of activated genes [43]. Of note, not all distant p53 binding sites showed the characteristic chromatin signature of enhancers: we also identified strong p53 peaks in distal regions of presumably condensed chromatin, devoid of H3K4me3, H3K4me1, or H3K27ac in unstressed conditions. These sites were high affinity sites, as supported by the identification of the consensus p53 motif, and did not overlap with any known regulatory regions in B cells (data not shown) [47]. It was previously demonstrated that p53 binds preferentially genomic regions with high nucleosome density and is able to induce nucleosome reorganization [46, 48]. Distal sites containing strong p53-REs and located in repressed chromatin were recently described to acquire H3K27ac and H4K16ac activation marks upon p53 binding and were proposed to act as “proto-enhancers” [24]. However, it was also observed that these sites have very low conservation scores due to the presence of repeated elements of viral origin [25]. Therefore, the functional nature of these sites is still unclear and requires further investigation.

p53 binding profiles were very similar in B and non-B cells (Figure 2F), indicating that the hypothesis of a ‘default set’ of p53 binding sites not influenced by the activating stimulus [10, 13, 16] holds true in different cell types and contexts. Despite the similarity in overall p53 binding profiles, the p53-dependent transcriptional changes were only in part coinciding (Figure 5D), implying a two-stage mechanism whereby p53 binding should be followed by a second layer of regulation, such as co-factors recruitment or post-translational modifications, to result in differential expression in diverse cell types.

The comparison of p53 binding and expression profiles, revealed that p53-dependent gene regulation could not be inferred simply from p53 binding nearby the TSS, as many p53-independent genes - induced by irradiation also in p53 knockout animals - showed a p53 binding site. Yet, a correlation could be drawn between sequence-specific binding and regulation: in particular, in line with previous reports [16], our data indicate that p53 binding to the unsplit p53-RE is associated with gene activation, but not repression. Of notice, however, p53-RE binding *per se* is insufficient to determine activation, suggesting that additional context-dependent signals contribute to the transcriptional response.

Our data do not exclude that p53 could still mediate direct (DNA binding-mediated) gene repression at sites without the p53 motif or containing the motif with a spacer, via either displacement of specific activators from promoters due to the presence of overlapping binding sites, the binding of p53 to “repression” response elements, or recruitment of co-repressors, such as histone deacetylases [49]. However, these models have been challenged by a recent meta-analysis of large-scale data, based on which it was concluded that p53 is solely an activator [50]. In line with an indirect repression mechanism, the repressed genes in our dataset were mainly involved in cell cycle progression and mitosis, whose down-regulation is most likely to be mediated through the induction of *Cdkn1a/*p21, resulting in the activation of repressive E2f4 complexes [51].

Altogether, our observations that the unsplit p53 motif as well as p53 binding intensity are coupled with the strongest p53-dependent gene induction suggest that these p53-bound DEGs represent the main effectors of the p53 response. Of note, these genes were activated in the two cell populations analyzed here, suggesting that they constitute the core of a cell type-independent p53 program. In line with this model, most of these genes were previously reported to be bound and regulated by p53 in different cell types and in response to different stress stimuli. Our data have significantly extended our understanding of this core p53 program and have confirmed its relevance in response to genotoxic stress in an intact organism.

## MATERIALS AND METHODS

### Mouse breeding and genotyping

The following mouse strains were used for this study: p53 KO (p53^Δ^) mice [52] and C57/Bl6 wild-type mice (Harlan). All animals were maintained on a C57/Bl6 background and bred to obtain the genotype combinations described in this paper.

Primers used for genotyping were listed below.

**Table:** 

Strain	Primers
p53 KO	AGCGTGGTGGTACCTTATGAG GGATGGTGGTATACTCAGAGC GCTATCAGGACATAGCGTTGG

Experiments involving animals were performed in accordance with the Italian law (D.lgs. 26/2014), which enforces Dir. 2010/63/EU (Directive 2010/63/EU of the European Parliament and of the Council of 22 September 2010 on the protection of animals used for scientific purposes).

### Primary mouse B and non-B cells

B cells were extracted as described. Spleens from C57/Bl6 wild-type or *Trp53^−/−^* mice were collected four hours after exposure to 7 Gy whole-body irradiation and from a control cohort of mice. Single-cell suspensions were obtained by pressing the spleens through nylon cell strainers and subsequent hypotonic lysis of red blood cells. To isolate B cells, we incubated single-cell suspensions with B220 MicroBeads (Miltenyi Biotech) and enriched them by magnetic cell sorting (MACS), according to the manufacturer instructions (Miltenyi Biotech). The column flow through was kept to represent the non-B cell population.

### Western blotting

5×10^6^ to 10×10^6^ cells were lysed with RIPA Buffer (50 mM Tris-HCl pH 8, 150 mM NaCl, 5 mM EDTA, 0.5% NP-40, 0.1% SDS, supplemented with protease inhibitors (Mini, Roche) and phosphatase inhibitors 0.2 mM Ortovanadate, 5 mM NaF) and sonicated. Cleared lysates were electrophoresed and immunoblotted with the indicated primary antibodies: p53 (NCL-p53-CM5p) from Novocastra laboratories, phospho-histone H2A.X (Ser139) clone JBW301 from Millipore and Vinculin (V9264) from Sigma. After incubation of the membranes with appropriate secondary antibodies, imaging was performed using an enhanced chemiluminescence (ECL) detection kit (Bio-Rad), followed by analysis with ChemiDoc XRS+ imaging system and Image Lab software (Bio-Rad).

### Flow cytometry: cell cycle and apoptosis analysis

To analyze cell cycle and apoptosis, 1×10^6^ live cells were resuspended in 1 ml of PBS and fixed by adding 2 ml of ice-cold absolute ethanol and kept at 4°C for at least 30 minutes. Cells were washed once with 1 ml of PBS 1% BSA and stained overnight with 1 ml 50 μg/ml propidium iodide and 250 μg/ml RNaseA at 4°C. At least 10,000 total events were analyzed by FACS. All the FACS data were acquired using a FACSCalibur machine (Becton Dickinson) and then analyzed by using FlowJo software (TreeStar).

### Chromatin Immunoprecipitation

Cells were processed as described above.

To minimize inter-individual variation we decided to process ChIP samples in pools of ten age- and sex-matched animals. For ChIP-Seq analysis of p53, lysates from 50×10^6^ B and non-B cells were immunoprecipitated with 10 μg p53 antibody (NCL-p53-CM5p - Novocastra laboratories). p53 antibody specificity was tested by ChIP-Seq analysis in total splenic cells from *Trp53^−/−^* mice four hours after 7 Gy of irradiation.

Cells were fixed by addition of 1% formaldehyde for 10 min. Fixation was stopped by addition of 0,125 M glycine. Cells were washed three times in PBS, resuspended in SDS buffer (50 mM Tris-HCl pH 8, 0,5% SDS, 100 mM NaCl, 5 mM EDTA, protease and phosphatase inhibitors) and stored at −80°C before further processing for ChIP. Cells were pelleted by centrifugation, and suspended in 4 ml of IP Buffer (100 mM Tris-HCl pH 8.5, 0.3% SDS, 1.7% Triton X-100, 100 mM NaCl, and 5 mM EDTA). Cells were disrupted by sonication with a Branson 250 sonicator, performing 5 cycles of 30 sec 30% amplitude, yielding genomic DNA fragments with a bulk size of 100-400 bp. 1 ml of diluted lysate was precleared by addition of 25 μl of blocked protein A beads (50% slurry protein A-Sepharose, Amersham; 0.5 mg/ml fatty acid-free BSA, Sigma; and 0.5 mg/ml tRNA, Sigma, in TE). Samples were immunoprecipitated overnight at 4°C with polyclonal antibodies. Immune complexes were recovered by adding 50 μl of blocked protein A beads and incubated for 4 hr at 4°C. Beads were washed with successive washes in 1 ml of Mixed Micelle Buffer (20 mM Tris-HCl pH 8, 150mM NaCl, 5mM EDTA, 5% (w/v) sucrose, 1% Triton X-100, and 0.2% SDS), Buffer 500 (50 mM HEPES pH 7.5, 0.1% (w/v) deoxycholic acid, 1% Triton X-100, 500 mM NaCl, and 1 mM EDTA), LiCl Detergent Wash Buffer (10 mM Tris-HCl pH 8.0, 0.5% (w/v) deoxycholic acid, 0.5% NP-40, 250 mM LiCl, and 1 mM EDTA), and TE (pH 7.5). DNA was eluted in TE 2% SDS and crosslink reversed by incubation overnight at 65°C. DNA was then purified by Qiaquick columns (Qiagen) and quantified using PicoGreen (Invitrogen). 2-10 ng ChIP DNA was prepared for HiSeq2000 sequencing with TruSeq ChIP Sample Prep Kit (Illumina) following manufacturer instructions. For ChIP-qPCR analysis, DNA was amplified in Real-time RT-PCR reactions with FAST SYBR Green Master Mix (Applied Biosystems).

### RNA extraction and analysis

Total RNA was purified onto RNeasy columns (Qiagen) and treated on-column with DNase (Qiagen). Complementary DNA (cDNA) was produced using the reverse transcriptase ImPromII (Promega). 10 ng of cDNA were used for Real-time RT-PCR reactions with FAST SYBR Green Master Mix (Applied Biosystems).

For RNA-Seq, total RNA from 10^7^ cells was purified using Trizol (Invitrogen), treated with Turbo DNase (Ambion) and purified with RNA Clean XP (Agencourt). 5 μg of purified RNA were then treated with Ribozero rRNA removal kit (Epicentre) and ethanol precipitated. RNA quality and removal of rRNA were checked with the Agilent 2100 Bioanalyzer (Agilent Technologies). Libraries for RNA-Seq were then prepared with the TruSeq RNA Sample Prep Kits v2 (Illumina) following manufacturer instructions starting from the RNA fragmentation step.

### DNase I hypersensitivity

Genome-wide sequencing of DNase I hypersensitive sites (DNase I seq) was performed as described [53, 54]. Briefly, apoptotic cells were removed by separation through a Ficoll gradient and live cells were washed with PBS. Pipetting in the following steps was performed with cut tips to avoid DNA breaks due to pipetting force. Cells were resuspended in buffer A (15mM Tris-HCl pH 8, 15mM NaCl, 60mM KCl, 1mM EDTA pH 8, 0.5mM EGTA pH 8, freshly supplemented with 0.5mM spermidine and 0.15mM spermine). An equal volume of lysis buffer (buffer A with 0.1% NP-40) was added and the cells were incubated on ice for 10 min. Nuclei were pelleted, washed once with buffer A and then resuspended at a concentration of 50×10^6^ nuclei per ml. Then 10^7^ nuclei were diluted with an equal volume of 2X DNase I reaction buffer (Roche). DNase I (Roche, 04716728001) was added at increasing concentrations (0, 100, 200, 300, 400, 500 U ml^−1^) and DNA was digested for 10 min at 37°C. An equal volume of Stop buffer (50mM Tris-HCl pH 8, 100mM NaCl, 0.1% SDS, 100mM EDTA pH 8, freshly supplemented with 0.5mM spermidine, 0.15mM spermine and 10 μg ml^−1^ of RNase A) was added. Samples were incubated at 55°C for 30 min (220 r.p.m. agitation). Then 0.2 μg ml^−1^ of proteinase K was added and samples were incubated at 55°C overnight (220 r.p.m.). DNA was extracted using a standard phenol-chloroform extraction protocol, dissolved in 100 μl of TE (55°C, 2 h). Then 300 ng of DNA of each digested sample was checked on an agarose gel for the appearance of a smear of slightly digested DNA. Small molecular weight DNA was purified using AMPure beads (Agencourt AMPure XP Reagent, A63881). The digested DNA samples (100 μl) were supplemented with 50 μl of AMPure beads, 150 μl of 20% PEG buffer (20% PEG8000, 2.5M NaCl) and incubated for 15 min at room temperature. Beads were separated on a magnet, washed twice with 80% ethanol and small molecular weight DNA was eluted in 100 μl of 5.5% PEG buffer. The eluted DNA was purified once more (20 μl of beads; 120 μl of 20% PEG buffer) and after washing eluted in 20 μl of H_2_O. DNaseI performance was checked by qPCR and samples for sequencing were selected based on the highest signal-to-noise ratio based on selected genomic regions (with 200-300 U ml^−1^ of DNase I). Chosen samples were size-selected on an agarose gel, small molecular weight DNA ( < 500 bp) was eluted from the gel with a Qiagen Gel purification kit according to the manufacturer's instructions. Up to 10 ng DNA was prepared for HiSeq2000 sequencing with TruSeq ChIP Sample Prep Kit (Illumina) following the manufacturer's instructions.

### NGS data filtering and quality assessment

ChIP-seq and RNA-seq NGS reads sequenced with the Illumina HiSeq2000 were filtered using the fastq_masker (setting the options to −q 20 −r N) tools of the FASTX-Toolkit suite (http://hannonlab.cshl.edu/fastx_toolkit/). Their quality was evaluated and confirmed using the FastQC application (http://www.bioinformatics.babraham.ac.uk/projects/fastqc/).

### Analysis of ChIP-seq data

ChIP-seq NGS reads were aligned to the mm9 genome through the BWA aligner [55] using default settings. Peaks were called using the MACS software (v1.4.1) [56], with the option ‘-p 0.00000001’, thus outputting only enriched regions with *p*-value < 10^−8^. Normalized read counts within a genomic region were determined as the number of reads per million of library reads (total number of aligned reads in the sequencing library). Peak enrichment was determined as log_2_(ChIP_w_/N_c_ - input_w_/N_i_), where ChIP_w_ is the read count on the enriched region in the ChIP sample, input_w_ the read count on the same region in the corresponding input sample, N_c_ is the total number of sequenced reads in the ChIP sample, and N_i_ is the total number of reads in the input sample.

### RNA-seq data analysis

RNA-Seq NGS reads were aligned to the mm9 mouse reference genome using the TopHat aligner (version 2.0.6) [57] with default parameters. In case of duplicated reads, only one read was kept. Read counts were associated to each gene (based on UCSC derived mm9 GTF gene annotations) using the featureCounts software [58] (http://bioinf.wehi.edu.au/featureCounts/) setting the options -T 2 -p -P. Absolute gene expression was defined determining RPKM as previously described [59], defining total library size as the number of reads mapping to exons only. Differentially expressed genes (DEGs) were identified using the Bioconductor [60] package DESeq2 [61] based on read counts, considering genes whose q-value relative to the control is lower than 0.05 and whose maximum expression is higher than RPKM of 1 in at least one replicate in the conditions considered. Quantification of relative levels of total mRNA was calculated on B and non-B cells RNA-Seq data from 4 *Trp53^+/+^* and 2 *Trp53^−/−^* mice in each condition.

### Other bioinformatic and statistical analyses

Bioinformatics and statistical analysis, including heatmaps, GeneOntology enrichment, and hierarchical clustering of RNA-Seq data were performed using R and Bioconductor packages [60].

### Motif search

Enriched motifs from the top 1000 p53 binding sites were determined *de novo* using MEME [37]. The inferred position weight matrix (PWM) described the p53 binding motif, composed by two decameric half sites. In order to account for the possibility of a spacer, an array of 15 PWMs was designed, containing a string of 1-15 uniformly distributed nucleotides between the two halves. The 600 bases flanking the p53 peaks summits were matched to the PWMs with FIMO [38]. When multiple copies of the p53 motif were identified, the peak was associated to the one with the lowest *p*-value, as calculated by FIMO.

### Primer Design and List of Primers

Primers for ChIP and mRNA analysis were designed using the primer design software Primer-BLAST [62] (Supplementary Table 2).

### Data access

We have uploaded the NGS datasets used in our paper onto the GEO archive under the accession number GSE71180.

## SUPPLEMENTARY MATERIAL TABLES




